# Primary Mediastinal B-Cell Lymphoma and [18F]FDG PET/CT: What We Learned and What Is New

**DOI:** 10.3390/hematolrep17030023

**Published:** 2025-04-28

**Authors:** Anna Giulia Nappi, Francesco Dondi, Achille Lazzarato, Lorenzo Jonghi-Lavarini, Joana Gorica, Flavia La Torre, Giulia Santo, Alberto Miceli

**Affiliations:** 1 Section of Nuclear Medicine, Interdisciplinary Department of Medicine, University of Bari “Aldo Moro”, Piazza Giulio Cesare 11, 70124 Bari, Italy; annagiulia.nappi@policlinico.ba.it; 2 Nuclear Medicine, ASST Spedali Civili di Brescia and Università degli Studi di Brescia, 25123 Brescia, Italy; 3 Nuclear Medicine Unit, ARNAS G. Brotzu, 09047 Cagliari, Italy; achille.lazzarato@aob.com; 4 Nuclear Medicine Department, IRRCS San Gerardo dei Tintori di Monza, 20900 Monza, Italy; lorenzomaria.jonghi-lavarini@asst-cremona.it; 5 Department of Radiological Sciences, Oncology and Anatomo-Pathology, Sapienza, University of Rome, 00161 Rome, Italy; joana.gorica@uniroma1.it; 6 Nuclear Medicine Unit, Department of Biomedical and Dental Sciences and of Morpho Functional Imaging, University of Messina, 98125 Messina, Italy; latorreflavia@gmail.com; 7 Department of Experimental and Clinical Medicine, “Magna Graecia” University of Catanzaro, 88100 Catanzato, Italy; giulia.santo@unicz.it; 8 Nuclear Medicine Unit, Azienda Ospedaliero-Universitaria SS. Antonio e Biagio e Cesare Arrigo, 15121 Alessandria, Italy; alberto.miceli@ospedale.al.it

**Keywords:** PMBCL, [18F]FDG, PET/CT, staging, restaging, diagnosis

## Abstract

Primary mediastinal large B-cell lymphoma (PMLBCL) is a rare and aggressive non-Hodgkin lymphoma (NHL), considered a specific entity with proper characteristics, therapies, and prognosis. First-line treatment is not unique, and subsequent strategies in case of disease persistence or relapse are the subject of debate and studies. In this scenario, [18F]FDG PET/CT plays a pivotal role both in characterizing the mediastinal mass, the main feature of PMLBCL, in staging, in restaging during therapy (interim PET), and at the end of treatment (EoT PET), to guide clinical management and give prognostic insights. The main issue with PMLBCL is distinguishing viable disease from residual fibrotic/inflammatory mass after therapy and, consequently, settling the next clinical strategy. Novel therapeutic approaches are ongoing and associated with the deepening of [18F]FDG PET/CT potentials as a principal tool in this context. In this review, we will explore PMLBCL from a Nuclear Medicine point of view to help clinicians in the management of these patients.

## 1. Introduction

Primary mediastinal large B-cell lymphoma (PMLBCL) is a rare and aggressive subtype of non-Hodgkin lymphoma (NHL), originating in the mediastinum and characterized by a diffuse proliferation of medium to large B-cells in association with the presence of sclerosis. Previously classified as a diffuse large B-cell lymphoma (DLBCL) subtype, it is now recognized as a separate entity in the Lymphoid Malignancies WHO classification, in relation to its molecular, histological, and clinical characteristics [1,2]. PMLBCL constitutes 2–3% of all NHL [1,3], and an annual incidence rate of 0.4 million has been derived in the US population from the Surveillance, Epidemiology, and End Results (SEER) database [4], based on more than 400 patients diagnosed between 2000 and 2012, predominantly adolescents and young adults [1]. PMLBCL mostly affects patients in the third and fourth decade of life, with a peak of incidence at 30–39 years old, with a prevalence in females over males (ratio: 1.5), especially in white subjects. The five-year survival rate is generally 85%, while among specific populations (Asians, Hispanics, etc.) it is about 70%. Prognosis is lower for patients aged ≥ 60 years old, with a higher estimated risk (3.5 times greater) compared with young adults (18–39 years). The late stage of disease, “other populations” groups, and socioeconomic status represent significant prognostic variables [4]. No specific risk factors have been identified; however, genetic susceptibility, immune disorders, obesity, infectious conditions, occupational exposures, and socioeconomic status would appear to be involved in the increased incidence of disease observed in recent decades [1,5,6,7].

Morphologically, PMLBCL is defined by a diffuse proliferation of a large B-cell population within the thymus, with polymorphic nuclei and with clear or slightly basophilic cytoplasm, sclerosis, and a compartmentalization degree characterized by a fibrotic reaction with reticulin fiber thin strands surrounding neoplastic cell clusters. PMLBCL expresses B-cell antigens such as CD19, CD20, CD22, CD79a, and CD45, but they do not express surface immunoglobulins. CD30 is expressed in ~80% of cases and MAL (myelin and lymphocyte) in ~70%, differing from DLBCL and classical Hodgkin lymphoma (cHL) rare occurrence, while CD3, CD10, and CD21 are typically negative. Abnormal expression of MAL protein could be implicated in the lymphomagenesis process and neoplastic growth control [8,9]. Transcription factors that often turn out to be positive include PAX5, OCT2, BCL6, PU1, IRF4, and BOB1. PMLBCL pathobiology depends on molecular processes involving REL, JAK-STAT, PDL1, PDL2, and NFkB. Most common PMLBCL chromosomal abnormalities involve Cr 9p24 (with a JAK2 dysregulation and abnormal expression, with PDL1 and PDL2 amplification) and Cr 2p16 with REL proto-oncogene duplication encoding for NF-kB. Another mechanism involved is CIITA rearrangement, an MHC class II gene transactivator with a limitation of tumor cell interaction with T-cells. MHC-II downmodulation and PDL1 and PDL2 overexpression are functional to PMLBCL survival in the thymic microenvironment. JAK-STAT pathway deregulation is involved in oncogene activation, tumor suppressor gene inactivation, abnormal cell proliferation, tumor growth, and metastasis [1,10,11,12,13].

In more than two-thirds of cases, PMLBCL appears with a large mass in the anterior mediastinum, often accompanied by local compressive symptoms such as dyspnea, cough (airway involvement and obstruction), and dysphagia. More than half of patients manifest with vena cava syndrome, which is characterized by the presence of thoracic and neck vein distension, facial and arm edema, and conjunctival swelling. One-third of patients present common B-symptoms (fever, night sweats, and weight loss) [14]. Other clinical features that may occur include pleural/pericardial effusions and elevated serum lactate dehydrogenase (LDH). This mediastinal tumor can be frequently over 10 cm (bulky mass), infiltrating the lung, chest wall, pleura, and pericardium (70–80%). The first manifestation can be both nodal and extranodal, but distant lymph node involvement is rare. Symptoms develop rapidly, and 80% of cases are diagnosed as stages I-II [1]. However, a quarter of patients present with advanced-stage disease. PMLBCL recurrence often occurs in the extranodal district, with liver, gastrointestinal tract, kidney, adrenal gland, ovary, and central nervous system (leptomeningeal or intraparenchymal disease) involvement, with relatively uncommon bone marrow spread (1–5% of cases) [15].

PMLBCL diagnosis is based on clinical, morphological (large anterior mediastinal mass), and immunophenotypic criteria. Sclerosis may constitute the dominant histological feature of the microscopic picture with high heterogeneity in tumor different areas. Diagnostic tissue samples should be, therefore, obtained by tumor mass percutaneous needle biopsy, mediastinoscopy, or by anterior mediastinotomy, or mini-thoracotomy [16]. The staging work-up is based on the Ann Arbor Staging System for Hodgkin and non-Hodgkin lymphoma. Diagnosis and staging are determined by history, physical examination, imaging studies, laboratory tests, initial biopsy, immunohistochemistry, and bone marrow aspirate [17]. The main imaging methods for PMLBCL diagnosis are represented by Computed Tomography (CT), Magnetic Resonance Imaging (MRI), and Positron Emission Tomography (PET). A mediastinal mass, a deviation of the tracheal axis with or without caliber narrowing, pleural/pericardial effusions, and unilateral hemidiaphragm elevation can be observed even on initial evaluation using X-ray imaging. However, CT is commonly used as a first-line imaging modality for detecting tumor extension. Namely, neck and chest CT scans allow us to identify the presence of posterior mediastinum or subcarinal region tumor masses, which represent less frequent PMLBCL localizations, as well as the superior vena cava syndrome with mediastinal vessel compression and venous thrombosis. Mediastinal masses usually show low-attenuation features on CT, including necrosis, cystic degeneration, and bleeding. Despite its significant role in staging, CT is less useful in recognizing residual lesions and monitoring treatment efficacy because of its limited role in discriminating between residual disease or post-treatment fibrotic reaction [18]. MRI can play a role in the definition of the intrathoracic extension of the disease, in assessing large vessels, as well as cardiac and chest wall involvement. Similarly, after treatment, but with scarce prognostic value, the residual tumor often shows a heterogeneous signal intensity on T2-weighted images, while high signal intensity on T2 or low signal intensity on T1 can be related to inflammation, necrosis, or active lymphoma. On the contrary, homogeneous hypointensity can be appreciated in fibrotic masses [18,19,20,21,22,23,24,25].

[18F]Fluorodesoxyglucose (FDG) PET/CT is a well-known diagnostic tool commonly used for FDG-avid HL and NHL. This imaging method is useful for characterizing lymphadenopathies or masses, for guiding biopsy, and for detecting extra-mediastinal tissues such as bone marrow involvement. Moreover, also in PMBCL, [18F]FDG PET/CT plays a major role in staging, and afterward, in monitoring response to treatments. Commonly, Revised Lugano classification and International Harmonization Project in Lymphoma, using a five-point visual scale, commonly known as the Deauville score (DS), are used. The DS allows us to compare the most intense uptake in a site of initial disease with the tracer uptake in the liver and in the mediastinal blood pool to define metabolic response as complete, partial, or progressive disease after treatments (for more details, see Supplementary Materials).

### Treatment Options for PMLBCL

First-line treatment choice plays an essential role in PMLBCL because of the limited efficacy of salvage therapy of recurrence/progressive disease. The major open issues regarding PMLBCL treatment deal with (i) which type of chemotherapy regimen to adopt as the first line (choice of a first- or third-generation chemotherapy protocol, including or not including Rituximab); (ii) the evaluation of high-dose protocol benefits; and (iii) the role of consolidation radiotherapy (RT) on the mediastinum, maintaining a balance between the fraction of cure (as high as possible) and the reduction in long-term morbidity (as much as possible) [3,26].

Generally, a higher rate of refractory/relapsed disease is observed in patients with PMLBCL compared to DLBCL treated with cyclophosphamide, doxorubicin, vincristine, and prednisone (CHOP) protocol [26]. In the pre-Rituximab era, PMLBCL patients showed higher response rates (with longer PFS and OS), when treated with third-generation chemotherapy (such as etoposide/methotrexate, doxorubicin, cyclophosphamide, vincristine, prednisone, and bleomycin—MACOP-B/VACOP-B) compared to CHOP protocol, as shown by several European studies [27,28]. In 2006, after the approval of Rituximab by the FDA, an improvement in outcomes in all DLBCL subtypes was shown by using the combined protocol (R-CHOP, DA-EPOCH-R, R-VACOP-B). After first-line treatment, consolidation RT can improve response rate and long-term survival in subjects with residual or recurrent mediastinal disease in this setting, despite possible long-term harmful effects due to radiation exposure, especially given the young age of these patients. Long-term harmful effects include the increased risk of second malignancy (particularly, breast cancer) and cardio-pulmonary damage, such as accelerated coronary artery disease [29]. Some studies which compared R-CHOP therapeutic protocol plus RT vs. DA-EPOCH-R regimen, without RT, showed similar efficacy between the two different therapeutic strategies. However, whether high-dose chemotherapy can replace RT remains to be addressed [30], and generally, the role of RT in PMLBCL is, anyway, a matter of debate. The reliability of consolidation use is undeniable, especially in PMLBCL with residual disease (DS > 3), possibly converting a PMR into CMR after chemotherapy [27]. Conversely, the use of RT after chemotherapy in patients with CMR remains uncertain and needs further study. Currently, the initial treatment of PMLBCL has no universally accepted standard of care or regimen.

As second-line treatment with R- dexamethasone, cytarabine and cisplatin/oxaliplatin (DHAP)/(DHAOX) is used for four cycles, plus carmustine/fotemustine, etoposide, cytarabine, and melphalan (BEAM/FEAM), followed by autologous stem cell transplant (ASCT) [31]. New therapeutic approaches are now under investigation and will be discussed in this review.

Further, histological confirmation before embarking on further aggressive salvage chemotherapy could be considered in some cases [32,33,34].

This comprehensive review aims to demonstrate and summarize the role of [18F]FDG PET/CT in every step of patient management, from staging to treatment response evaluation, in order to guide decision-making regarding the best personalized treatment option, and to explore the role of [18F]FDG PET/CT as a prognostic marker for patients affected by PMLBCL. Finally, an overview of the question under study/future perspectives in the evolving field of PMLBCL will be given.

## 2. [18F]FDG PET/CT in PMLBCL

### 2.1. Diagnosis

Even if histological results are mandatory to confirm diagnosis, there is increasing interest in the potential role of [18F]FDG PET/CT in characterizing mediastinal masses before biopsy to guide the clinician in the diagnostic process.

One of the main concerns, in fact, is the ability of [18F]FDG PET/CT to differentiate PMBCL from other mediastinal entities. In this scenario, some studies aimed to assess the reliability of different PET parameters to distinguish PMBCL from thymic epithelial tumors (TETs) (Table 1). In a 2020 study, Zhu and colleagues, in a cohort of 136 patients pathologically diagnosed with TETs or PMBCL, showed that all metabolic parameters extracted from baseline [18F]FDG PET/CT significantly differed between groups; specifically, patients with lymphomas were younger and had higher SUVmean, SUVmax, TLG, and MTV values than patients with TETs [35].

Recently, Yan et al. confirmed these data in a similar retrospective study with 304 patients, finding that lymphomas were significantly associated with younger patient age, higher LDH level, larger tumor size, and higher SUVmax compared to TETs (*p* < 0.001). Moreover, the accuracy of age, LDH, tumor size, and SUVmax in predicting lymphoma was 84.8%, 67.8%, 85.2%, and 78.3%, respectively. The combination of the four above parameters could improve the predictive accuracy to 89.1% [36].

An interesting work regarding the use of baseline [18F]FDG PET/CT in PMBCL was performed by Alkhawtani et al. [37]. They included in their study a total of 56 patients (42 with mediastinal HL and 14 with PMBCL) to evaluate the ability of PET/CT metrics to differentiate HL from PMBCL, which can both present with an anterior mediastinal tumor mass. Lesion-to-liver SUVmax ratio and lesion-to-liver SUVpeak ratio were significantly higher in PMBCL than in HL (*p*-value < 0.001). Furthermore, higher lactate dehydrogenase (LDH) value, larger tumor diameter, and more frequent necrosis were all elements that helped to distinguish PMBCL from HL, reflecting the more aggressive tumor biology of the former compared to the latter. AUC for lesion-to-liver SUVmax ratio and lesion-to-liver SUVpeak ratio were 0.875 and 0.874, with optimal cut-off values of 7.12 (94.9% sensitivity and 64.3% specificity) and 11.45 (97.4% sensitivity and 64.3% specificity), respectively [37].

To differentiate the most common subtype of bulky mediastinal lymphomas, Abenavoli et al. enrolled 117 patients, of which 80 had HL, 29 had PMBCL, and 8 had gray zone lymphoma (GZL), and combined radiomics with metabolic texture analysis (TA) to quantitatively estimate intratumor heterogeneity. The authors found that SUVmax and TLG showed the highest values in the PMBCL group, and MTV was higher in PMBCL and GZL compared to the HL group, as expected in line with the most aggressive biological behavior of PMBCL. In addition, metabolic heterogeneity is also a remarkable feature of malignancy, and texture analysis showed a higher value of heterogeneity and entropy in PMBCL than in the HL group. Referring to the GLZM matrix, the Zone-Length Non-Uniformity, which measures the variability of the intensity values of the gray-level in the image, showed higher values in PMBCL. Machine learning (ML) algorithms combined with radiomics could be a robust histological-based tool in discriminating lymphoma subtypes [38].

### 2.2. Staging

It has generally been reported that [18F]FDG PET/CT performed before any treatment in lymphoma can give fundamental prognostic information, and PMBCL does not make any exception [39]. Staging [18F]FDG PET/CT could identify the minority of PMBCL patients who experienced chemorefractory disease associated with poor outcomes. The first to evaluate the role of staging [18F]FDG PET/CT in PMBCL patients were Ceriani et al. [40], based on the International Extranodal Lymphoma Study Group (IELSG) 26 study [41]. All the 103 patients included had undergone subsequent treatment with rituximab plus anthracycline-containing regimens (R-CHOP or R-CHOP like, R-VACOP-B or R-MACOP-B), and 93 of them had also undergone consolidation RT on the mediastinum. A significantly shorter PFS and overall survival (OS) were associated with an increase in MTV (PFS analysis: HR 1.36; 95%CI: 1.16–1.61, *p* < 0.001; OS analysis: HR, 1.39, 95%CI: 1.08–1.77, *p* = 0.009) and TLG (PFS analysis: HR 1.40; 95%CI: 1.25–1.58, *p* < 0.001, OS analysis: HR 1.47, 95%CI: 1.22–1.76, *p* < 0.001), but not with an increase in SUVmax. Only TLG demonstrated statistical significance for both OS (*p* < 0.001) and PFS (*p* < 0.001) in multivariate analysis. Long-term outcomes were significantly better for subjects with low TLG, and this parameter showed an extremely high negative predictive power, since no deaths and only one relapse were reported among patients with low baseline TLG values. TLG was confirmed as the strongest independent predictor of PMBCL outcomes for both OS (*p*-value < 0.0027) and PFS (*p*-value < 0.0001), to risk-stratify patients that could benefit from treatment intensification [40].

This evidence, which emerged from an IELSG-26 study for patients treated with non-EPOCH-based treatment, was confirmed by Pinnix et al., who retrospectively enrolled 65 patients with newly diagnosed PMBCL and who were treated with a DA-EPOCH regimen [24]. Of baseline PET/CT semiquantitative parameters (SUVmax, MTV, and TLG), the area under the curve (AUC) was significant for TLG (0.756; *p*-value 0.006), and the cut-off value of 3941.4 g proved optimal for classifying patients at low and high risk of progression, with sensitivity and specificity of 83% and 70%, respectively. Machine learning-derived thresholds for both MTV and TLG showed promising results in predicting disease progression. Subsequent multivariate analysis confirmed TLG as the only reliable prognosticator (HR, 7.879; *p*-value 0.049). Finally, if elevated baseline TLG was combined with an end-of-treatment Deauville score of 4 to 5, the sensitivity improved to 83% in predicting disease progression.

Similarly, Liu et al. confirmed the powerful prognostic role of baseline MTV and TLG in predicting worse PFS in 26 PMBCL patients that received DA-R-EPOCH and consolidation RT. The authors reported cut-off values of 500 for MTV (*p* = 0.002) and 2500 g for TLG (*p* = 0.023), respectively, correlated with worse PFS [42].

However, Zhou et al., in their retrospective study enrolling 166 patients treated with different chemotherapy regimens (R-CHOP, R-EPOCH, and R-HCVAD), focused on the prognostic value of different clinical–genetical features, and considered SUVmax as the only PET parameter. The authors showed that patients with SUVmax > 11.6 at diagnosis had the worst OS (*p*-value 0.021), and most of them were treated with the R-EPOCH regimen [43].

Going beyond PET semi-quantitative parameters, Ceriani et al. [44,45] showed interest in studying tumor heterogeneity, a complex phenomenon that reflects variable phenotype profiles, genome instability, and epigenetic variation that has been associated with chemoresistance and treatment failure. [18F]FDG PET/CT may show metabolic intratumoral heterogeneity (MH), previously associated with poor prognosis in solid tumors [46,47,48,49]. The authors evaluated the prognostic role of the MH of baseline [18F]FDG PET/CT images in the known IELSG-26 study cohort. MH was defined as the percentage of tumor volume, with SUV above a certain threshold, plotted against threshold values varying from 25% to 100% of SUVmax, excluding necrotic areas with very low or absent FDG uptake [44]. The AUC was a quantitative index of tracer uptake heterogeneity, with lower values corresponding to increased heterogeneity [45]. MH distribution was also evaluated by estimating the coefficient of variation in the intratumoral FDG uptake, calculated as the standard deviation of the SUV divided by the SUVmean of the segmented lesion. MH values did not show a significant relationship with other baseline PET semiquantitative parameters (SUVmax, MTV, TLG). The receiver-operating characteristic (ROC) analysis of AUC identified an optimal cut-off of 0.45 to discriminate patients with disease progression or relapse, with a sensitivity of 69% (95%CI: 39–91%) and specificity of 72% (95%CI: 62–81%). In this setting, subjects with progression or relapse had significantly higher MH than those with complete remission (*p* = 0.012). PFS was significantly longer for patients with low MH (*p* < 0.0001). Elevated MH (HR 12.8, 95%CI: 3.3–49.9, *p* < 0.001) and elevated TLG (HR 46.5, 95%CI: 5.8–373.8, *p* < 0.001) were found to be independent prognosticators for PFS, so they were combined in a prognostic model for PFS: patients with both high TLG and high MH had poorer outcomes (HR: 31.2, 95%CI: 9.9–97.8, *p* < 0.001). MH may be an additional biomarker in PMBCL, suggesting that combining it with TLG in a prognostic model may improve PPV and may adequately screen high-risk patients needed for more intensive treatment [45].

Due to the prognostic importance of volumetric PET parameters, but the low reproducibility of the results, the same study group investigated the most reliable method and threshold to accurately estimate MTV, evaluating the accuracy in predicting PFS and OS in the IELSG-26 study cohort [50]. In this study, the authors compared methods for metabolic volume segmentation by applying three thresholds (FT25% = 25%, FT41% = 41% of the SUVmax, and FT2.5 = an absolute SUV value of 2.5) and by means of a region growing (RG) algorithm [48]. In PMBCL patients, characterized by a dominant bulky mediastinal lesion, the FT25% provided the most accurate estimation of the actual volume. Patients with low MTV had significantly longer PFS and OS, regardless of the method used, but the optimal cut-offs for MTV to predict PFS and OS were method-related. The four methods predicted disease progression with similar NPV (95–98%), but the highest PPV (45%) was for FT25%, which provided the best model to identify patients with the worse outcome. These results confirmed the relationship between the most accurate estimation of the actual volume of the lesions and the best risk classification [50].

In conclusion, volumetric parameters (MTV and TLG), particularly TLG, could be considered powerful prognostic biomarkers to predict PFS and OS, as shown by all reported studies’ results, and the combination of these parameters with MH could improve the risk-adapted strategies in PMBCL patients. Table 2 summarizes the characteristics of studies included in this section.

### 2.3. Early Treatment Response Assessment—iPET

The use of interim [18F]FDG PET/CT (iPET) has been widely proven to have an excellent NPV and accurate prognostic insights, especially in HL (with the subsequent creation of multiple assessment scales, e.g., DS) [51,52,53,54] as well as in DLBCL [55,56,57,58,59]. Nevertheless, some controversy about the role of iPET remains, for example, in advanced-stage disease patients [60,61]. This issue is also debated in PMBCL patients, in which the prognostic significance of iPET remains unclear due to the scarcity of available data, the relative rarity of the PMBCL subtype, and the small sample size enrolled in the few studies currently available, whose data should be used with caution due to a lack of a standardized method of interpretation of iPET results in this context (mainly change in SUV value—ΔSUV, or a 5-point scale DS) [62].

In 2014, Avigdor and colleagues retrospectively analyzed the value of iPET in 30 PMBCL consecutive patients receiving Rituximab-containing chemotherapy and who underwent iPET before starting the seventh cycle of R-VACOP-B or after the third cycle of R-CHOP21. On the basis of visual assessment, a negative iPET was able to efficiently predict excellent outcome, since a negative iPET was reported in 16/30 subjects (50%) who remained in complete response at the end of therapy, with a single exception. Furthermore, a positive iPET identified subjects with poor prognosis and, in particular, 14/30 patients (47%) had positive findings: nine of them had CMR, one had PMD, and four had PMR at the end of treatment. The 5-PFS was 94% (95%CI 62–99%) and 57% (95%CI 28–78%) for negative and positive iPET, respectively (*p* = 0.015). Overall, 70% of the cohort had the outcome predicted by iPET, which confirmed a high NPV (94% and 100% for R-VACOP-B and 86% for R-CHOP) but a poor PPV (43% and 30% for R-VACOP-B and 75% for R-CHOP) [63]. Interestingly, this low value of PPV could be predicted by persistent inflammatory response and fibrosis, persisting even after a successful treatment regimen, therefore mimicking the presence of residual neoplastic mediastinal mass [34,64]. Based on the visual assessment, negative iPET efficiently predicted an excellent outcome; indeed, iPET was negative in 16/30 patients (53%), and all remained in complete response at the end of therapy, with only one relapse. Positive iPET partly identifies patients with poor prognosis; indeed, iPET was positive in 14/30 patients (47%) and 9/14 had CMR, while 4/14 had PMR and 1/14 had PMD at the end of therapy. Further, 5-year PFS was 94% (95%CI 62–99%) for negative iPET and 57% (95%CI 28–78%) for positive iPET (*p* = 0.015). Overall, iPET predicted treatment outcome in 70% of the patients, confirming a high NPV (94% and 100% for R-VACOP-B and 86% for R-CHOP) but a PPV of only 43% (30 for R-VACOP-B and 75% for R-CHOP) [63]. The low PPV could be explained by the inflammatory fibrosis that persisted even after successful treatment, mimicking residual mediastinal mass [34,64].

Cheah et al.’s study emphasized the high NPV (87–88%) of iPET, irrespective of the method of interpretation, while positive iPET was not predictive of inferior PFS, confirming the low PPV for relapse (12.5–15%) [34].

In a retrospective study, Lazarovici et al. directly compared positive iPET results with histological analysis consisting in a surgical debulking of the residual mediastinal mass or a CT-guided needle biopsy. iPET was performed after the fourth cycle of immunochemotherapy in 36 PMBCL treated by combining an anti-CD20 monoclonal antibody with CHOP or CHOP-like chemotherapy. Applying visual analysis subsequently reassessed using DS, iPET was positive in 17 (47%) patients, of which 3 (17%) has DS ≤ 3 and 14 (82%) had DS 4, and was negative in 19 (53%) patients, of which 14 patients (74%) had DS ≤ 3 and 5 (26%) had DS 4. All positive groups underwent histological restaging, which revealed inflammatory necrosis and fibrosis in 15/17 patients, silicosis in 1 patient, and persistent disease in only 1 patient. Given the frequent iPET positive results in mediastinum, the authors concluded that iPET may be considered a false positive in 94.1% of cases with a very low PPV (16.6%), meaning it does not reflect the persistence of active disease in the vast majority of PMBCL cases, regardless of using the International Harmonization Project (IHP) criteria, ΔSUV, or DS [62]. However, in a comment published by Qin et al., the authors underlined the absence of interim DS 5 patients in the aforementioned cohort. Thus, they investigated the role of iPET in 49 PMBCL patients (44.9% received R-CHOP, and 55.1% DA-EPOCH-R). Subjects with interim DS 5, of which 5/10 patients also had histological confirmation, had poor treatment response and shorter survival; indeed, the overall response rate (ORR) was 30.0% (3/10), a value significantly lower than in those patients with interim DS 1–3 (96.8%, *p* < 0.001) or DS 4 (87.5%, *p* = 0.025), respectively. Moreover, the 2-year PFS value was 20% for interim DS 5, which was significantly shorter than interim DS 1–3 (93.4%, *p* < 0.001) or DS 4 (69.3%, *p* = 0.012); OS was 67.7% for interim DS 5, shorter than interim DS 1–3 (95.8%, *p* = 0.003). No differences in ORR, PFS, or OS were observed between patients with interim DS 1–3 and DS 4, so these were categorized as DS 1–4 with a good outcome; indeed, none of them experienced disease progression or relapse during follow-up. These results are more encouraging regarding the role of iPET in effectively identifying PMBCL patients with poor clinical outcomes, based on the high PPV of interim DS 5, that should be candidates for further treatment [64].

Therefore, the prognostic significance of iPET in PMBCL is still debatable and remains, moreover, an open issue, because of the inconsistency of results obtained in clinical studies and reported in the literature. Whilst the NPV is still excellent, the relatively low PPV and specificity [62,63] and high false positive results were frequently related to benign lymphoid reactive or follicular hyperplasia, sarcoid-like granulomatosis, thymic hyperplasia or rebound, and fibrosis [65]. To explain such a phenomenon, some authors speculate that the overexpression of the glucose transporter GLUT1 could play a role in the persistent mediastinal [18F]FDG avidity [34,66], and that the addition of rituximab to chemotherapy could produce an inflammatory response that could cause increased [18F]FDG uptake [67]. Since it is commonly accepted that most positive iPET results correspond to an actual residual inflammatory fibrosis and not to a viable tumor, it is a widespread opinion that good outcomes can still be achieved with chemotherapy even in most patients with a positive iPET that thus have frequently no impact on clinical decision-making [62].

To improve the specificity of iPET, numerous studies state that a histological confirmation of the residual mass should be mandatory, and, therefore, therapeutic changes for PMBCL should not be made solely based on iPET results. Larger multicentric collaborations in randomized clinical study trials with large multicenter collaborations are still needed to understand the natural evolution of PET results in PMBCL across different treatment time-points, to validate the prognostic value of iPET and to achieve a common agreement on the most reliable and powerful predictor, the iPET interpretation method [34,62,68,69]. Characteristics of included studies are summarized in Table 3.

### 2.4. End-of-Treatment Response Assessment—EoT PET

Currently, [18F]FDG PET/CT is incorporated into the therapeutic decision-making for ensuring tailored chemo- and radiotherapy strategies in PMBCL patients [41]. To overcome the PMBCL tendency to recur, mediastinal RT is incorporated in different therapeutic strategies, since it is capable of converting partial to complete response, and of consolidating disease control in case of complete response [70]. However, various authors sustained that PET-based response after different therapeutic regimens should be considered as a reference, limiting RT indication eventually to PET-persistent disease patients [71,72,73] (Table 4).

In several studies with different immunochemotherapy protocols, the authors shared a strategy of administering RT to PET-positive patients, while it was omitted for clearly PET-negative patients or based on the decision of a treating physician [74,75]. Similarly, in a recent publication by Held et al., in a subgroups analysis of the UNFOLDER Trial, 131 PMBCL patients were randomized in a 2 × 2 factorial design to R-CHOp-21 vs. R-CHOP-14, and additive RT vs. observation, demonstrating a significant superior 3-year event-free survival after RT, without impact on PFS and OS. The authors concluded that RT seemed not to be required for all patients, and PET should be used to guide clinical decisions. RT might be spared for patients achieving a PET complete response after an R-CHOP-based regimen, while poor responders with PET-positive residual mass (DS 4–5) might benefit from RT [76].

This PET-adapted strategy significantly reduces (−64%) the number of patients who received consolidative RT without affecting outcome [77]. However, it is not fully clarified whether RT might be avoided solely based on a negative PET scan [78]. A different PET-based strategy was adopted by Shemmari et al. that administered consolidation RT in PET-negative patients after R-CHOP, while PET-positive patients were treated with salvage chemotherapy followed by ASCT [31].

Finding true positive and true negative PET, however, is the crucial issue in PMBCL scenarios, with respect to other lymphomas.

In this context, [18F]FDG PET/CT surely helps to detect small mediastinal residual lesions important for better RT planning, even if a high incidence of false positive findings is reported after rituximab-base regimens, as mentioned before. A longer time interval from the last treatment cycle and the EoT PET might reduce the false positive rate, but increase the risk of missing the opportunity to cure persistent disease with RT. Although invasive, a new biopsy of remnant tissue for a histological confirmation should be discussed and considered in some cases before starting subsequent aggressive salvage chemotherapy too quickly [32,33,34].

The use of EoT PET imaging to predict patient outcomes is of considerable interest in PMBCL patients, especially thanks to the application of response assessment criteria useful for standardizing physician interpretation. PET response was initially established according to the IHP criteria, based on visual analysis alone [79]. In recent years, response assessment was preferentially based on the internationally validated DS [80,81]. This scale quantifies uptake in residual tumor masses to better differentiate active lymphoma from fibrotic mass [33]. Based on these criteria, the aforementioned prospective IELSG-26 study provided the first validation of Lugano classification in defining treatment response in a PMBCL cohort, to be used for patient risk stratification and treatment adaptation. Unlike other forms of [18F]FDG-avid lymphoma, a DS-3 category was considered borderline, depending on the timing of evaluation and the clinical context [82]. In the IELSG-26 study, a worse PFS (99% vs. 68%) and OS (100% vs. 83%) were observed for DS 4–5 patients compared to DS 1–3 [41,80]; therefore, patients with EoT DS > 3 should be considered at higher risk of relapse and candidates for further treatments [72,83,84]. Different authors emphasized the excellent NVP (95–100%) of EoT PET negative scan (DS 1–3) [34,85]. From these premises, the cut-off point for PET positivity was moved from the mediastinal blood pool uptake (DS 3) to liver uptake (DS 4), to increase PPV (from 18% to 32%), and to ensure a clearer distinction between risk subgroups in terms of PFS and OS [24,31,41,80,83].

Although a better PPV was observed for the EoT PET compared to iPET, showing a reduction in the DS from interim to EoT [78], the EoT PET PPV result was still limited, given that post-immunochemotherapy inflammation commonly occurred. In a subanalysis of the IELSG-26 trial focusing on the irradiation response assessment, persistent [18F]FDG uptake after RT seems likely to reflect a post-treatment inflammatory reaction rather than an active disease, not necessarily requiring salvage therapy, confirming the unsatisfactory specificity for PET-positive scan (DS ≥ 4) even after RT [84]. Probably, Deauville criteria have some limitations in correctly discriminating minimal refractory disease from inflammatory reaction. In contrast with these results, the IELSG-37 trial suggested a prudential cut-off of DS 3, considering those with DS 3 as PET-positive patients, but with uncertainty on the appropriate management [80].

A strategy to reduce the false positive rate in EoT PET could be the combination of visual analysis provided by DS with semiquantitative parameters, including SUVmax, SUVmean, MTV, and TLG [41,72,83,86,87]. Particularly, the combination of DS 5 with SUVmax > 5 was strongly associated with a worse PFS [43,83]. Vassilakopoulos et al. speculated that most DS 4 patients could register false positive findings, especially if the uptake is relatively low (SUVmax < 5), suggesting an RT-sparing approach in this case. The initiation of further salvage chemotherapy in patients with persistent [18F]FDG-uptake (even if SUVmax ≥ 10), but DS < 5, was discouraged because these patients frequently showed a long-term remission without further treatment [86,87,88], supporting Filippi et al.’s results, which showed an excellent outcome without relapse in all DS 4 patients [83].

IELSG37 was a clinical trial with the objective of assessing whether RT could be omitted in PMBCL patients with CMR (DS 1–3) after treatment with chemo-immunotherapy. This was a randomized, non-inferiority protocol, and its results have been recently reported. The total cohort was composed of 545 subjects with PMBCL; 268 of them reached CMR after therapy and were then randomly assigned to observation or RT (130 and 136 patients, respectively). PFS at 30 months was 96.2% and 95.8% in the observation and RT arm, respectively (stratified HR 1.47; 95%CI 0.34–6.28), while OS was similar in both the groups (99% each). The findings of this study suggested, therefore, that patients with CMR after chemo-immunotherapy could safely omit consolidation RT [89].

The optimal treatment strategy for PMBCL patients is still controversial, and more aggressive therapeutic strategies have been developed, reasonably omitting mediastinal RT [90]. In a National Cancer Institute (NCI) phase 2 prospective study, a dose-intense DA-EPOCH regimen, especially when used with rituximab (DA-R-EPOCH), demonstrated excellent outcomes (5-year OS 97%) and tumor control, with consolidation RT administration only in 4% of patients [73,78,91]. The choice of DA-EPOCH regimen as a front-line option in PMBCL patients was supported by the higher complete metabolic response rate in comparison with R-CHOP (84% vs. 70%), despite a similar OS and PFS [73,92]. The prognostic value of EoT PET in the context of DA-R-EPOCH therapy was demonstrated by an inferior 5-year event-free survival (92% vs. 80%) in PET-positive patients (DS 4–5). Analogously to other therapeutic regimens, EoT PET showed a poor PPV (17%) and a high false positivity rate due to post-therapy inflammatory reaction, as histologically demonstrated by Dunleavy et al. [91]. Probably, [18F]FDG PET/CT alone may not be accurate enough to determine the presence of residual disease after DA-R-EPOCH therapy [91]. However, Giulino-Roth and colleagues sustained a more pronounced impact of EoT PET in predicting outcomes after DA-R-EPOCH with a sensitivity, specificity, PPV, and NPV of 76.5%, 83.3%, 41.9%, and 95.7%, respectively [93].

A risk-adapted strategy, including PET semiquantitative data as well, would also be desirable in the DA-R-EPOCH response assessment [41]. EoT PET findings, including higher SUVmax, DS 4–5, and a CT-documented residual mass, were associated with inferior PFS and increased risk of progression [24]. Moreover, the combination of pre- and post-treatment PET findings may better identify high-risk patients. In Pinnix et al.’s study, the prognostic model combining high baseline TLG (>3941.4) with EoT DS 5 ensures sensitivity, specificity, PPV, and NPV of 50%, 98%, 86%, and 90%, respectively [24]. Several authors suggested integrating Deauville criteria, with MTV and TLG being good predictors in DA-R-EPOCH response assessment, improving PPV (20%) and NPV (98%) [43]. 

The integration of the DS with SUVmax could help to adequately select patients who need consolidative mediastinal RT after DA-R-EPOCH [92,94]. In particular, with a careful approach, RT should be considered for higher-risk patients with DS 4–5 and SUVmax ≥ 5.4 [80]. Alternatively, Melani et al. suggested that PET-negative patients, but also PET-positive ones up to DS-4 after DA-R-EPOCH, can be managed with follow-up only, since they would not benefit from consolidation RT [43].

Serial PET imaging could effectively help to discriminate residual disease from post-treatment inflammatory reaction, and should be considered for selecting patients who require consolidation RT. An overall SUVmax decrease across serial scans is associated with non-progressive disease, while an SUVmax increase is associated with treatment failure. Even if limited by low tumor volume following therapy, MTV and TLG appeared similar, but not superior, to SUVmax [43].

Mediastinal RT could markedly impact on a better complete response rate in the PMBCL population treated with more aggressive regimens, as the third-generation MACOP-B and VACOP-B [71]. EoT PET evaluation may represent a useful tool at the time of RT planning, sparing mediastinal irradiation in PET-negative patients (DS 1–3) [71,73,95]. Even after the MACOP-B/VACOP-B regimen, Zinzani et al. described false positive results, underlining the importance of serial PET scans to reveal a gradual decrease in SUV value in responder patients [96].

Hayden et al. chose a similar PET-adapted approach to guide the consolidative RT administration in PMBCL patients treated with R-CHOP/R-ICE (rituximab, ifosfamide, carboplatin, etoposide)/DA-R-EPOCH [78]. An observational strategy was adopted for EoT PET-negative patients (DS 1–3) with a superior 5-year time to progression (TTP) and superior OS compared to positive ones (DS 4–5) (5-year TTP 91% vs. 68%; 5-year OS 97% vs. 87%), recommended to receive RT. Considering DS 4 patients, it remains uncertain whether it could represent a false positive post-treatment inflammatory state [97].

EoT PET’s prognostic role was also documented after the introduction of new treatment protocols, known as GMALL/B- ALL/NHL (alternating immunochemotherapy with intermediate-dose methotrexate for six cycles) [98], in a Rituximab-Lymphomes Malins B (LMB)-based chemotherapy regimen [99], and also in transplantation patients who experienced disease progression following front-line anthracycline-based chemotherapy and who underwent salvage chemotherapy. Namely, an inferior OS was observed in EoT PET non-responder patients [100].

An open issue in this context is, surely, the best timing for performing EoT PET with respect to the end of therapy. For instance, 4 to 6 weeks after the end of chemotherapy is a widely accepted time-point, but no focused studies investigating different time-points have been conducted.

**Table 4:** Characteristics of included studies about EOT [18F]FDG PET/CT.

Year	Authors	N. Patients	Therapy	PET Parameters and Findings
2014	Martelli et al. [41]	125	Rituximab and anthracycline-containing chemoimmunotherapy	DS. Performed using liver uptake as the cut-off for PET positivity (from DS 3 to 4), better discrimination is found between high and low risk of failure, with 5-year PFS of 99% versus 68% (*p* = 0.001) and 5-year OS of 100% versus 83% (*p* = 0.001).
2021	Casadei et al. [71]	151	MACOP-B regimen with or without Rituximab	Visual analysis. Patients with a negative PET scan after MACOP-B had complete response and did not undergo RT.
2016	Pinnix et al. [72]	97	R-CHOP, or R-HCVAD, or R-EPOCH with or without RT	DS and SUVmax. Patients with DS 4–5 and SUVmax > 5.4 after immunochemotherapy were at high risk of progression or relapse.
2015	Zinzani et al. [73]	74	R-MACOP-B	Visual analysis. A PET-guided RT approach leads to reducing the use of RT.
2019	Chan et al. [30]	124	DA-EPOCH-R, R-CHOP	Visual analysis. PET provides information to evaluate the value of chemo- and RT.
2008	De Sanctis et al. [70]	92	MACOP-B	Visual analysis. PET may be effective in therapy response assessment
2022	Karakatsanis et al. [74]	339	R-CHOP-14, R-CHOP-21	DS. RT was limited to PET-positive patients with DS 3–5. The use of RT for DS 1–2 patients was at the discretion of the treating physician.
2023	Held et al. [76]	131 PMBCL patients (UNFOLDER trial)	R-CHOP-21, R-CHOP-14, RT for bulky disease ≥7.5 cm or extralymphatic involvement (82 patients)	Visual analysis. RT should be applied to poor responders that showed partial response with residual lesion at EoT PET (DS 4–5).
2021	Vassilakopoulos et al. [75]	332	R-CHOP	DS. In case of PET-negative patients, RT should be avoided. Salvage therapy in responding patients with positive PET should be avoided in absence of progression or multifocal disease.
2021	Morgenstern et al. [77]	56	DA-EPOCH-R R-CHOP/R-ICE	DS. PET provides information to evaluate the value of chemo- and RT.
2016	Goldschmidt et al. [78]	47	R-CHOP-RICE	DS. EoT DS ≤ 3 predicts favorable outcomes.
2014	Shemmari et al. [31]	20	R-CHOP	Visual analysis. Salvage chemotherapy and BEAM autologous marrow transplant was performed after R-CHOP in PET-positive subjects. Better OS and PFS was demonstrated in patients who had undergone PET scan compared to those receiving RT on the bases of CT imaging.
2011	Tai et al. [32]	19	R-CHOP	Visual analysis. RT planning and stage migration based on PET improved survival outcome.
2015	Yang et al. [33]	28	Rituximab	Visual analysis. PET results impact on the choice of post-induction treatment. Patients with PET-evaluation were more likely to receive RT alone (28.6% vs. 0%).
2015	Cheah et al. [34]	28	R-CHOP, CHOP-Q14, DA-EPOCH-R	SUVmax. PET has excellent NPV but limited PPV due to the high frequency of positive scans. A negative PET is an excellent predictor of outcome. Residual metabolically active masses after treatment should be biopsied to confirm viable lymphoma prior to salvage therapy.
2013	Filippi et al. [80]	37	Rituximab-based chemotherapy	DS. Almost 50% of PMBCL patients demonstrate residual disease at EoT PET. DS 5 patients are at high risk of progression and death, and they might be candidates for intensified programs.
2016	Filippi et al. [83]	51	R-CHOP and R-CHOP-like chemotherapy	DS. Metabolic response after chemo-immunotherapy proved to be a strong prognostic factor for PFS after radiotherapy. The use of PET for patient risk stratification allowed us to identify a subgroup of patients at high risk of progression/relapse after radiotherapy.
2017	Ceriani et al. [84]	88	R-CHOP, R-CHOP-like, R-VACOP-B, R-MACOP-B	DS and Lugano classification. PET can identify patients at higher risk of progression after RT. The prognostic value of the Lugano classification criteria was confirmed in the response assessment after RT. Patients who achieved a complete metabolic response (DS ≤ 3) all remain progression-free at 5 years. Patients with DS 4 also had excellent outcomes, suggesting that they do not necessarily require additional therapy, since the residual FDG uptake may not reflect persistent lymphoma.
2015	Nagle et al. [85]	27	R-CHOP	Visual analysis. Negative iPET and EoT PET identified patients who achieve long-term remission. Both positive iPET and EoT PET need to be better defined.
2018	Pinnix et al. [24]	65	DA-R-EPOCH	MTV, TLG, and DS. A model combining baseline TLG and EoT DS identified patients at increased risk of progression.
2016	Vassilakopoulos et al. [86]	106	R-CHOP	DS, Lugano classification, and SUVmax. Based on PET/CT results, no salvage chemotherapy and ASCT in subjects who respond to R-CHOPOmission of RT in a considerable amount of PET-negative subjects.
2021	Vassilakopoulos et al. [87]	182	R-CHOP	DS. The decision to start further salvage chemotherapy in the absence of progression of disease in conventional imaging should not be triggered by the persistence of positive PET/CT with DS < 5 after consolidative RT, should not trigger the initiation of further salvage chemotherapy in the absence of conventionally defined progression disease RT.
2020	Zhou et al. [43]	166	R-HCVAD, R-EPOCH, R-CHOP	SUVmax. EoT PET SUVmax has correlations with survival outcome.
2012	Vassilakopoulos et al. [88]	76	MACOP-B, R-CHOP	Visual analysis. RT can be safely omitted in selected patients based on a negative EoT PET.
2022	Velasques et al. [90]	93	DA-EPOCH-R, R-CHOP/R-CHOEP	Lugano criteria. PET may be an alternative to prevent patients from the long-term deleterious effects associated with RT.
2013	Dunleavy et al. [91]	69	DA-EPOCH, DA-EPOCH-R	SUVmax. PET alone is not accurate for tumor response assessment, since it showed a poor PPV = 17%. The shrinkage of residual mediastinal mass continued for 6 months, suggesting that inflammatory cells might account for FDG uptake.
2018	Shah et al. [92]	132	R-CHOP, DA-EPOCH-R	PET may serve as a disease assessment tool to determine the need for EoT RT.
2017	Giulino-Roth et al. [93]	156	DA-R-EPOCH	DS. Negative EoT PET was associated with improved EFS (95.4% vs. 54.9%, *p* < 0001). Patients with a positive EoT PET scan have an inferior outcome.
2018	Melani et al. [101]	93	DA-R-EPOCH	DS. Serial PET imaging distinguished EoT PET-positive patients without treatment failure, thereby reducing unnecessary RT by 80%.
2021	Jain et al. [94]	43	R-EPOCH	DS. The patients with single-site residual disease on EoT PET underwent localized RT.
2017	Broccoli et al. [95]	98	MACOP-B, R-MACOP-B	DS. RT was spared in those patients with a negative PET corroborative of a complete response.
2009	Zinzani et al. [96]	45	R-MACOP-B, R-VACOP-B	SUVmax. Seven patients who had PR and presented with an ambiguous PET pattern turned out to be associated with false positives later on.
2020	Hayden et al. [97]	113	R-CHOP	DS. The use of a PET-adapted approach reduces RT in the majority of patients.
2022	Romejko-Jarosinska et al. [98]	124	GMALL/B-ALL/NHL protocol	DS. EoT PET was predictive for outcome: 5-year OS and PFS were 96% and 94% in negative subjects, and 70% and 70% in positive subjects (*p* = 0.004 for OS, *p* = 0.01 for PFS).
2022	Dourthe et al. [99]	42	Rituximab-Lymphomes Malins B-based chemotherapy	Visual analysis. PET provides information to evaluate the value of chemo- and RT.
2018	Vardhana et al. [100]	60	Platinum-based chemotherapy, ICE chemotherapy, with or without Rituximab	Visual analysis. PET provides information to evaluate the value of chemo- and RT.
2019	Zinzani et al. [102]	30	Nivolumab, brentuximab vedotin	SUVmax and DS. DS 1–3 was associated with complete response.
2024	Yousefirizi et al. [103]	50	R-CHOP	Metabolic heterogeneity and radiomic analysis. Delta PET/CT radiomic features showed the most predictive performance, especially in specific sub-regions closer to the center of the tumor, outperforming both baseline and EoT features only and the use of PET features alone in predicting progression/relapse.

Acronyms: DS, Deauville score; EoT, end of treatment; RT, radiotherapy; NPV, negative predictive value; PPV, positive predictive value.

## 3. PMLBCL: What Is New?

New therapeutic approaches are, now, under investigation, given that the rituximab-based strategy could be insufficient in a small subset of PMBCL patients with highly aggressive disease. Therapies based on specific cellular targets consisting of membrane proteins, signal transduction pathways, programmed death ligands, etc., have been developed.

CAR-T cell therapy, for example, is one of the most promising new treatment options. This novel approach is based on the redirection of immune system T-cells against antigens expressed by tumor cells, and in the specific case of PMBCL, T-cells bind to an antigen expressed on the B-cell surface (Anti-CD19 CAR-T).

In a study performed at the NCI, the authors evaluated autologous anti-CD19 CAR T-cell therapy in 15 patients with treatment refractory CD19-positive B-cell malignancies, including 4 patients with R/R PMBCL. Of these four patients, treated with at least three prior lines of therapy, two (50%) obtained a CR, one (25%) was found to have stable disease, and another (25%) was not evaluable. Of note, both patients with a CR had ongoing responses at 12 and 22 months, respectively [104]. A retrospective study reported the real-world outcomes of 33 patients with R/R PMBCL who received axicabtagene ciloleucel (axi-cel) off-trial [105]. Patients had an average of three prior lines of treatment (range, 1–9). In the intention-to-treat population, the ORR was 76%, with a 67% CR rate. 2-PFS was 64% (95%CI 49–84%) and 2-OS was 78% (95%CI 64–96%). However, Grade 3 or higher cytokine release syndrome was seen in 6% of patients, and grade 3 or higher neurological toxicity in 27% of patients. The Italian prospective CART-SIE evaluated the efficacy and safety of axi-cel in 70 patients with R/R PMBCL, and 190 patients with other R/R large B-cell lymphomas, after at least two prior lines of treatment [106]. The ORR was 78% for patients with R/R PMBCL, with 50% obtaining a CR. Further, 12-month PFS was 62% (95%CI 51- 75%), and 12-month OS was 86% (95%CI 8–95%), both significantly better than patients with other R/R large B-cell lymphomas. Similar outcomes for axi-cel in R/R PMBCL beyond second-line treatment were observed in a GLA/DRST (German Lymphoma Alliance and the German Registry for Stem Cell) registry study [107]. Although the majority of patients enrolled in the four landmark CD19 CAR T-cell trials (ZUMA-1/axicabtagene ciloleucel, ZUMA-7/axicabtagene ciloleucel, TRANSCEND-NHL-001/lisocabtagene maraleucel, TRANSFORM/lisocabtagene maraleucel) were diagnosed with R/R DLBCL, a small number of patients with R/R PMBCL were included [108,109,110,111]. Responses were observed in all four trials, including durable remissions; however, due to the limited number of patients with R/R PMBCL, responses were grouped together. Importantly, both the ZUMA-7 and TRANSFORM trials evaluated CD19 CAR T-cell therapy as second-line treatment for R/R large B-cell lymphomas, and the authors compared this to standard care treatment (two or three cycles of investigator-selected salvage chemo-immunotherapy followed by HDT/ASCR). Both trials revealed improved response rates, EFS, and OS in patients with R/R large B-cell lymphomas treated with CD19 CAR-T cell therapy [110,111]. Based on the results of ZUMA-7, axi-cel was approved by the FDA in April of 2022 for adult patients with large B-cell lymphoma, including PMBCL, which is refractory to first-line chemo-immunotherapy or relapses within 12 months of first-line chemoimmunotherapy. Likewise, in June of 2022, the FDA extended the indication for lisocabtagene maraleucel (liso-cel) to include adults with large B-cell lymphoma, including PMBCL, who have refractory disease or relapse within 12 months of first-line chemo-immunotherapy based on the results of a TRANSFORM trial. A phase II trial is currently evaluating the combination of pembrolizumab plus anti-CD19 CAR-T cell therapy (either axicabtagene ciloleucel or lisocabtegene maraleucel) in patients with PMBCL with refractory disease or after two prior lines of therapy (NCT05934448). Other trials in different phases investigate CAR-20/19 T-cells (NCT04186520), sequential treatment of CD19 CAR NK- and 7x19CAR T-cells (NCT06464861), CD30.CAR T-cells (NCT04526834), WZTL-002 CAR-T cells (NCT06486051), CD79b-19 CAR T-cells (NCT06026319), P-CD19CD20-ALLO01 allogeneic CAR T-cells (NCT0601462), and cemacabtagene ansegedleuce (allogeneic) CAR T-cells (NCT06500273) for the treatment of R/R PMBCL.

A hallmark of PMBCL and cHL is, furthermore, the deregulation of the JAK-STAT pathway positively regulating PDL1 and PDL2 expression. PDL1 and PDL2 increased expression favor PMBCL survival in the thymic microenvironment. Antibodies directed against PD1 (Pembrolizumab) or PDL1 and JAK2 inhibitors (Ruxolitinib) could interfere with neoplastic cell survival mechanisms [74]. These checkpoint inhibitors have shown efficacy in relapsed/refractory disease (R/R PMBCL) patients, leading to FDA approval in PMBCL patients after two therapy line failures [97,112,113]. However, the results so far have been disappointing. A pilot phase II trial evaluating the JAK2 inhibitor ruxolitinib for the treatment of patients with R/R PMBCL (*n* = 6) resulted in rapid progression in patients [114].

Bispecific antibodies are a novel class of T-cell redirecting drugs and are among the most promising new immune-based strategies for B-cell lymphomas to date, including PMBCL. Few patients with PMBCL are now under investigation with these new agents with encouraging results, for example, with Glofitamab and Epcoritamab, both CD20 × CD3 bispecific antibodies [115,116,117].

In 2012, the Food and Drug Administration (FDA) approved Brentuximab vedotin (BV) for relapsed/refractory HL and anaplastic large cell lymphoma (ALCL) treatment, an anti-CD30 antibody highly expressed in this disease. This immunotherapy is under investigation even for PMBCL CD30+, although it expresses CD30 in a lower percentage. Few data are, for now, available regarding the use of BV in PMBCL. In a single-arm phase II trial consisting of 15 patients (53% with advanced stage disease, and 74% refractory to the most recent treatment), only two of them achieved PR, yielding an ORR of 13.3%. Consequently, the study was closed prematurely due to the low ORR at the mid-term evaluation [9]. Conversely, in a recent multicenter study, the combination of nivolumab with Brentuximab vedotin in a group of 30 rrPMBCL reached an ORR of 73% with a 37% CR rate per investigator and an ORR of 70%, with a 43% complete metabolic response rate per independent review. This favorable response rate was documented by serial PET scans to confirm complete metabolic response (DS 1–3) [102].

This evidence could pave the way for the introduction of new combined therapies to exploit the best treatment options for all PMBCL patients.

In this varied treatment landscape of PMBCL, even more complex, more precise imaging biomarkers to assess therapy response are required [74].

In this scenario, the conventional International Prognostic Index (IPI) has limited utility, given the young age of patients and the confined to the mediastinum disease [118]. As shown, similarly to other forms of lymphomas, [18F]FDG PET/CT is increasingly used to monitor treatment response and guide clinical decisions in PMBCL with DS and Lugano classification that have standardized [18F]FDG PET/CT interpretation, improving the management of patients, and providing prognostic insights, too [82,101,119,120,121].

The availability of an increasing number of biological agents, such as ICI, also requires flexibility in the interpretation of the recommendations to account for their biologic or immunomodulatory properties. Namely, tumor flare/pseudoprogression may occur during the first 2–3 weeks after the start of treatment and is characterized by a rapid, self-limited increase in the size and FDG uptake of the disease as an expression of transient and massive immune recruitment at the cancer site. Conversely, some patients could experience hyper-progression characterized by real tumor overgrowth and poor prognosis. In 2016, the Lymphoma Response to Immunomodulatory Therapy Criteria (LYRIC) were proposed, representing an adaptation of the Lugano classification for the evaluation of lymphoma after immune-based treatment. The LYRIC introduced the concept of an indeterminate response (IR)—instead of progression—to address such lesions until a biopsy or subsequent imaging, after 12 weeks, confirming true disease progression status [122].

Recently, emerging studies have demonstrated the utility of PET radiomics in further improving a tailored approach to treatment choice and EoT PET predictive power [97]. Metabolic heterogeneity could be extracted from PET images and could be an interesting radiomic potential marker, given the demonstrated chemoresistance and poor outcome of patients characterized by high intralesional heterogeneity [45]. Delta radiomics, capturing feature changes over time, could offer potential in evaluating treatment response, predicting outcome and improving clinical decision-making, with increased robustness compared to single time-point radiomics features. Intensity and textural features were extracted from EoT PET images in a Yousefirizi et al. study, and Delta PET/CT radiomic features showed the most predictive performance, especially in specific sub-regions closer to the center of the tumor, outperforming both baseline and EoT features only, and the use of PET features alone in predicting progression/relapse [103].

Even if IELSG-37 has demonstrated that supplemental RT on the mediastinum can be omitted in patients with a negative EoT PET/CT after chemo-immunotherapy without impacting on patient outcomes, an unmet need to early identify which patients with a positive EoT PET/CT would benefit from RT remains. It is possible that circulating tumor DNA (ctDNA) might have a role in the early identification of these patients in the future, as studies have shown a high PPV of ctDNA to predict relapse. In an interesting study, ctDNA monitoring was conducted in sixteen cases of enrolled patients, and fifteen of them showed undetectable ctDNA after a median of two (range from one to five) cycles, and one refractory case presented with durable positive ctDNA. Among four relapse cases, three had prior detectable ctDNA half a month earlier before imaging manifestations of relapse [123]. In another study, after one cycle of chemotherapy, three of four patients who reached CR at EoT already had undetectable ctDNA. One patient with positive ctDNA after one cycle needed RT to convert to CR. All the CR evaluations by PET-TC who had available ctDNA data presented undetectable ctDNA (*n* = 9) [124]. In a recent study, ctDNA exhibited high concordance with tissue samples and genomics characterization. Moreover, ctDNA fluctuation could reflect treatment responses and indicate relapse before imaging diagnosis [125]. Even if PMBCL is rare in children, emerging works suggest the use of whole-body MRI as an alternative tool in staging lymphomas [126]. A systematic review of seven studies reported that in the overall stage of disease, whole-body MRI showed excellent sensitivity (~ 95.8%) and a diagnostic odds ratio of 1.19, meaning that disease on whole-body MRI had a nearly equal likelihood of being positive as PET-CT. For extranodal staging, whole-body MRI showed both excellent sensitivity and specificity (~ 90%; 97.4%, respectively), as well as area under the SROC curve (0.913; partial AUC 0.875). However, general specificity in staging is still low [126].

Recently, a new PET radiotracer—[^68^Ga]Ga-Pentixafor, a chemokine receptor—has been developed for its expression on the surface cell of both solid and hematologic diseases such as lymphoma. Albano et al. performed a systematic review of the current literature in this setting, showing that [^68^Ga]Ga-Pentixafor PET can be a useful tool for the staging and treatment response evaluation, with a better diagnostic performance than [^18^F]FDG PET [127]. However, specific studies with [^68^Ga]Ga-Pentixafor PET and PMBCL patients have not yet been published.

## 4. Conclusions

Currently, PMBCL is a particular and specific entity in the panorama of lymphomas, with its own challenges and management. Different treatment lines are currently in use, with recent novel approaches that have increased PFS and OS in these patients.

This review shows how [18F]FDG PET/CT is crucial and plays a singular role in the initial diagnostic pathway to characterize the typical mediastinal mass and properly stage the patient, and that Interim PET and EoT PET are essential in evaluating the response to treatment, and, above all, distinguishing between residual disease and fibrotic/inflammatory remnants, which can lead to a change of therapy or an RT consolidation.

Given the heterogeneity and availability or choice of treatments, this review aims to resume progress in this field and evaluate how [18F]FDG PET/CT plays a central role in leading therapies, and in giving prognostic insights, too. For example, as shown a DS 1–3, or even DS 4 -EoT, [18F]FDG PET/CT can also lead to the avoidance of consolidation RT without any repercussions on survival outcomes. Patients with higher baseline MTV or a TLG at staging time-points have worse outcomes, suggesting an intensification therapy strategy. However, it is mandatory to underline the fact that different thresholds for these parameters have been reported in different studies.

Some limitations derived from the characteristics of the papers included in the review could clearly affect our findings. First, some of these studies are characterized by small and heterogeneous cohorts, with different types of therapeutic schemes. In addition, different semiquantitative metrics without strict standardization and external cross validation are utilized in the manuscripts included in this review. It is well known that many factors can influence semiquantitative parameters and radiomics, limiting their reproducibility. Finally, most of the papers had a retrospective design or lacked randomization. Based on these facts, no meta-analysis of the data retrieved could be performed.

Considering the peculiarities of this subtype of lymphoma, as well as its rarity, some aspects of functional imaging serving PMBCL remain to be investigated. Radiomics and artificial intelligence will, surely, help in this context soon. Even if data in the literature are still emerging, the role of these new tools could be critical in addressing several outstanding open issues. Routine integration of radiomics and AI will be fundamental to collect and analyze huge amounts of data, going beyond the human eye, to direct clinical management and predict outcomes more precisely.

Prospective randomized trials with [18F]FDG PET/CT-adapted strategies are necessary to further investigate this tool as a reliable disease biomarker in this particular clinical context. Furthermore, trials exploring new therapeutic approaches, such as CAR-T or combination therapies, with more patients involved, are necessary to verify the real impact of this new treatment.

## Figures and Tables

**Table 1:** Characteristics of included studies on [18F]FDG PET/CT at diagnosis.

Year	Authors	N. Patients	PET Parameters and Findings
2020	Zhu et al. [35]	136 (65 TET and 71 PMBCL)	Patients with lymphoma were younger and had higher SUVmean, SUVmax, TLG, and MTV values than patients with TETs.
2024	Yan et al. [36]	304 (60 with lymphoma, 244 with TET)	Lymphoma was significantly associated with younger patient age, higher LDH level, larger tumor size, and higher SUVmax compared to TETs (*p* < 0.001).
2018	Alkhawtani et al. [37]	56 (42 with mediastinal HL and 14 with PMBCL)	Lesion-to-liver SUVmax ratio and lesion-to-liver SUVpeak ratio were significantly higher (*p* < 0.001) in PMBCL than in HL. Larger tumor size and more frequent necrosis may help discriminate PMLBCL from HL (*p* = 0.001).
2023	Abenavoli et al. [38]	117 patients (80 HL, 29 PMBCL, 8 GZL)	SUVmax, SUVmean, SUVpeak, MTV, TLG, and texture analysis were measured or performed. SUVmax and TLG were significantly higher in PMBCL, intermediate in GZL, and lowest in HL. MTV was significantly higher in PMBCL and GZL compared to HL. Higher values of heterogeneity and entropy were detected in PMBCL. Zone-Length Non-Uniformity showed higher values in PMBCL.

Acronyms: TET, thymic epithelial tumor; HL, Hodgkin lymphoma; PMBCL, primary mediastinal B-cell lymphoma; LDH, Lactate dehydrogenase (LDH); SUVmax, standardized uptake value; MTV, metabolic tumor volume; TLG, total lesion glycolysis; l; GZL, gray zone lymphoma.

**Table 2:** Characteristics of included studies on [18F]FDG PET/CT at staging.

Year	Authors	N. Patients	Therapy	PET Parameters and Findings
2015	Ceriani et al. [40]	103 PMBCL patients (IELSG-26 study)	R-CHOP or R-CHOP-like RE-VACOP-B or R-MACOP-B; consolidation mediastinal RT (93 patients)	SUVmax, TLG, and MTV were included. Elevated MTV and TLG were significantly associated with worse PFS and OS, but not SUVmax.
2021	Liu et al. [42]	26 PMBCL patients	DA-EPOCH (11 patients) and Radiotherapy (17 patients)	SUVmax, SUVmean, MTV, TLG. MTV ≥ 500, and TGL ≥ 2500 were correlated with worse PFS (*p* = 0.002 and *p* = 0.023, respectively).
2020	Zhou et al. [43]	166 PMBCL patients	R-CHOP, R-EPOCH, R-HCVAD, RT (85 patients), SCT (31 patients)	SUVmax > 11.6 was associated with poor prognosis.
2018	Pinnix et al. [24]	65 PMBCL patients	DA-R-EPOCH	SUVmax, SUVmean, MTV, TLG. MTV, and TLG were powerful prognosticators in predicting progression.
2018	Ceriani et al. [45]	103 PMBCL patients (IELSG-26 study)	R-CHOP, R-CHOP-like, R-VACOP-B, R-MACOP-B, RT (93 patients)	SUVmean, SUVmax, MTV, TLG, and MH. MH and other parameters did not show any significant relationship. The AUC cut-off of 0.45 was optimal to discriminate patients with disease progression. PFS was longer for patients with low MH (*p* < 0.0001). Elevated MH and elevated TLG were independent prognosticators for PFS, so they were combined in a prognostic model for PFS: patients with both high TLG and high MH had poorer outcomes (*p* < 0.001).
2019	Ceriani et al. [50]	103 PMBCL patients (IELSG-26 study)	Not applicable	Comparison was made between methods for MTV segmentation by applying three thresholds (FT25%, FT41%, and FT2.5) and a region growing (RG) algorithm. FT25% provided the most accurate estimation of the actual volume. Patients with low MTV had significantly longer PFS and OS, regardless of the method used. The four methods predicted disease progression with similar NPV (95–98%), but the highest PPV (45%) was for FT25%, which provided the best model to identify patients with the worst outcome.

Acronyms: IELSG-26, International Extranodal Lymphoma Study Group; PMBCL, primary mediastinal large B-cell lymphoma; SUVmax, standardized uptake value; MTV, metabolic tumor volume; TLG, total lesion glycolysis; PFS, progression free survival; OS, overall survival; MH, metabolic heterogeneity.

**Table 3:** Characteristics of included studies about interim [18F]FDG PET/CT.

Year	Authors	N. Patients	Timing iPET	Therapy	PET Parameters and Findings
2014	Avigdor et al. [63]	92 PMBCL patients, of which 30 had iPET	iPET: before starting the seventh cycle of R-VACOP-B or after the third cycle of R-CHOP21	R-VACOP-B, R-CHOP21	Visual analysis. Mid-PET uptake was used to identify different PFSs; however, they had relatively low PPVs.
2017	Lazarovici et al. [62]	36 PMBCL patients	iPET: after the fourth cycle of immunochemotherapy	Anti-CD20 monoclonal antibody with CHOP or CHOP-like chemotherapy, dose-dense ACVBP, ASCT (nine patients), RT (seven patients)	Visual analysis, DS, and SUVmax were performed or measured. iPET false positive results were found in 94.1% of cases with a PPV of 16.6%, regardless of using visual analysis, SUVmax, or DS
2021	Qin et al. [64]	49 PMBCL patients	iPET: 3 weeks after three cycles	R-CHOP (22 patients), DA-EPOCH-R (27 patients), RT (14 patients)	For DS, iPET was useful in identifying PMBCL patients with poor clinical outcomes, based on the high PPV of interim DS 5, which should be a candidate for further treatment.
2015	Cheah et al. [34]	28 PMBCL patients, of which 23 ha iPET	iPET: 8 patients after two cycles, 13 patients after three cycles, and 2 patients after four cycles of chemotherapy	R-CHOP, DA-EPOCH-R, Hyper-CVAD ± R, RT s	Visual analysis and DS (DS ≥ 4 considered positive) were used. High NPV (87–88%) of iPET was detected, while positive iPET was not predictive of inferior PFS, confirming the low PPV for relapse (12.5–15%).

Acronyms: PMBCL, primary mediastinal B-cell lymphoma; iPET, interim PET; SUVmax, maximum standardized uptake value; DS, Deauville score.

## Data Availability

Data supporting the reported results can be found using public scientific databases.

## Supplementary Materials

The revised Lugano classification and the International Harmonization Project in Lymphoma supporting information can be downloaded at https://www.mdpi.com/article/10.3390/hematolrep17030023/s1.
