# Supplementary material for: Primary Mediastinal B-Cell Lymphoma and [18F]FDG PET/CT: What We Learned and What Is New
Source: Hematol Rep. 2025 Apr 28;17(3):23. doi: 10.3390/hematolrep17030023 (PMC12101282; doi:10.3390/hematolrep17030023)
Supplement: Supplementary file 1 [file hematolrep-17-00023-s001.zip › hematolrep-3536337-supplementary.pdf]

Supplementary Materials

[18F]FDG PET/CT response assessment

A revised Lugano classification and the International Harmonization Project in Lymphoma, using a five-point visual scale (Deauville Score - DS), are currently employed to evaluate treatment response.
The 5-PS scores the most intense uptake in a site of initial disease, if present, as follows:

- 1. No uptake;
- 2. Uptake ≤ mediastinum;
- 3. Uptake > mediastinum but ≤ liver;
- 4. Uptake moderately higher than liver;
- 5. Uptake markedly higher than liver and/or new lesions;
- X. New areas of uptake unlikely to be related to lymphoma.

International Conference on Malignant Lymphomas Imaging Working Group categorized responses as follows: negative PET/CT scan (DS 1-3) could represent a complete metabolic response (CMR); a significant FDG uptake reduction compared with baseline (DS 4-5) may represent a partial metabolic response (PMR) during the course of treatment; and finally, the absence of metabolic response corresponds to a DS of 4-5 at the end of treatment with no significant change compared to baseline. Stable metabolic disease (SMD) or progressive metabolic disease (PMD) includes a DS of 4-5 with new FDG-avid foci (>1.5 cm for nodal lesion and ≥ 1.0 cm for extranodal lesions) or significantly increased FDG uptake from baseline in a lymphoma treated with standard therapy [19–23].
In addition to DS, semi-quantitative parameters such as total lesion glycolysis (TLG) and metabolic tumor volume (MTV), might also be sensitive indicators for predicting progression-free survival (PFS) and overall survival (OS) [24,25].

**Modality**	**Clinical Application**	**Complete Response**	**Partial Remission/ Partial Response**	**Stable Disease/ No Response**	**Progressive Disease**
FDG PET/CT	FDG-avid lymphoma (including Hodgkin lymphoma and diffuse large B-cell lymphoma)	Complete metabolic response: score of 1, 2, or 3 in nodal or extranodal sites with or without a residual mass	Partial metabolic response: score of 4 or 5 with reduced uptake compared with baseline and residual mass(es) of any size	No metabolic response: score of 4 or 5 with no obvious change in FDG uptake	Score 4 or 5 in any lesion with an increase in intensity of uptake from baseline and/or new FDG-avid foci consistent with lymphoma
